# The Plasma NAD^+^ Metabolome Is Dysregulated in “Normal” Aging

**DOI:** 10.1089/rej.2018.2077

**Published:** 2019-04-23

**Authors:** James Clement, Matthew Wong, Anne Poljak, Perminder Sachdev, Nady Braidy

**Affiliations:** ^1^ Betterhumans Inc., Gainesville, Florida.; ^2^ Centre for Healthy Brain Ageing, University of New South Wales, School of Psychiatry, Sydney, Australia.; ^3^ Mark Wainwright Analytical Centre, University of New South Wales, Sydney, Australia.; ^4^ School of Medical Sciences, University of New South Wales, Sydney, Australia.; ^5^ Neuropsychiatric Institute, Euroa Centre, Prince of Wales Hospital, Sydney, Australia.

**Keywords:** NAD^+^, nicotinamide, aging, plasma, biomarker

## Abstract

Nicotinamide adenine dinucleotide (NAD^+^) is an essential pyridine nucleotide that serves as an electron carrier in cellular metabolism and plays a crucial role in the maintenance of balanced redox homeostasis. Quantification of NAD^+^:NADH and NADP^+^:NADPH ratios are pivotal to a wide variety of cellular processes, including intracellular secondary messenger signaling by CD38 glycohydrolases, DNA repair by poly(adenosine diphosphate ribose) polymerase (PARP), epigenetic regulation of gene expression by NAD-dependent histone deacetylase enzymes known as sirtuins, and regulation of the oxidative pentose phosphate pathway. We quantified changes in the NAD^+^ metabolome in plasma samples collected from consenting healthy human subjects across a wide age range (20–87 years) using liquid chromatography coupled to tandem mass spectrometry. Our data show a significant decline in the plasma levels of NAD^+^, NADP^+^, and other important metabolites such as nicotinic acid adenine dinucleotide (NAAD) with age. However, an age-related increase in the reduced form of NAD^+^ and NADP^+^—NADH and NADPH—and nicotinamide (NAM), N-methyl-nicotinamide (MeNAM), and the products of adenosine diphosphoribosylation, including adenosine diphosphate ribose (ADPR) was also reported. Whereas, plasma levels of nicotinic acid (NA), nicotinamide mononucleotide (NMN), and nicotinic acid mononucleotide (NAMN) showed no statistically significant changes across age groups. Taken together, our data cumulatively suggest that age-related impairments are associated with corresponding alterations in the extracellular plasma NAD^+^ metabolome. Our future research will seek to elucidate the role of modulating NAD^+^ metabolites in the treatment and prevention of age-related diseases.

## Introduction

In the last decade, there has been growing interest in the role of redox active nucleotides in the metabolism.^1^ The significance of pyridine nucleotide coenzymes, such as nicotinamide adenine dinucleotide (NAD^+^) and its phosphorylated form NADP^+^, as main electron transfer molecules and substrates for over 700 oxidoreductase enzymes is undebated.^2^ We and others have previously demonstrated that disturbances in the redox balance, for example, following exposure to chronic oxidative stress, often represents an important component of the pathobiology of cell loss in cardiovascular and neurodegenerative diseases.^3,4^ Exogenous stressors, such as overfeeding, starvation, alcohol ingestion, or drug treatment can alter the intracellular redox status of these coenzymes.^5^

NAD^+^ represents one of the most important coenzymes in the hydride transfer reactions.^6^ NAD^+^ is the precursor of the pyridine nucleotide family, including NADH, NADP^+^, and NADPH, and is the end product of tryptophan metabolism via the kynurenine pathway.^7^ It has been well established that NAD^+^ is a substrate for major dehydrogenase enzymes involved in nutrient catabolism, including alcohol and lactate dehydrogenase reactions.^8^ As well, NADH, which is the reduced form of NAD^+^, preferentially provides electrons to power mitochondrial oxidative phosphorylation. Apart from its roles in fuel utilization, NAD^+^ also serves as an exclusive substrate for the nuclear repair enzymes poly(adenosine diphosphate [ADP] ribose) polymerases (PARP). PARPs are a family of enzymes that are activated by double- or single-stranded DNA breaks in DNA, and are thought to promote DNA repair by the ADP-ribosylation of histones and other nuclear proteins.^9^ NAD^+^ is also a substrate for the enzyme NAD^+^ glycohydrolases (CD38) that leads to the production of cyclic ADP-ribose, a calcium efflux effector.^10^ NAD^+^ has also been shown to be the sole substrate for a new class of NAD-dependent histone deacetylase (“HDAC”) enzymes known as sirtuins.^11^ Increasing histone acetylation is associated with age-related pathologies, whereas gene silencing by upregulation of sirtuins has been shown to extend lifespan in yeast and small organisms.^12^ HDACs are also being found to interact with a variety of nonhistone proteins and to thereby change their function, activity, and stability by post-translational modifications.

Accurate determination of the NAD^+^ metabolome is of major interest due to its potential association with cognitive decline, including AIDS dementia complex,^13–15^ cancer,^16–18^ aging, and a plethora of age-related disorders. Recently, nicotinic acid adenine dinucleotide (NAAD), an intermediate of NAD^+^ synthesis from nicotinic acid (NA) via the NAD^+^ salvage pathway, has been shown to increase following ingestion with niacin.^19^ This finding suggests that increased NAD^+^ anabolism by supplementation with NAD^+^ precursors not only increases the accumulation of by-products of NAD^+^ catabolism (such as ADP-ribose and N-methyl-nicotinamide [MeNAM]), but also stimulates retrograde synthesis of NAAD and nicotinic acid mononucleotide (NAMN). However, the mechanism responsible for this elusive biochemical reaction is yet to be identified.

Given the significance of the NAD^+^ metabolome in a multitude of biological processes, accurate quantification of its concentration and redox state in plasma and tissue is essential for better understanding of important biochemical processes, and determining the metabolic state of organisms in response to treatment with various compounds and disease states. We and others have shown that the NAD^+^:NADH ratio varies between 1 and 10 in catabolic tissue of “physiologically” aged female Wistar rats, and human subjects.^3,4,20^ As NAD^+^ also serves as an oxidative agent in some biochemical processes such as fatty acid oxidation, glycolysis, and citrate cycle, changes to the NAD^+^:NADH ratio may also represent an indicator of alterations in metabolic processes and several diseases including multiple sclerosis. In 2011, we were the first to prove that NAD^+^ is an essential factor in the aging process in major declining levels of catabolic tissue such as the brain, heart, lung, liver and kidney of rats, and in human pelvic tissue.^3,4,20^ Increased NAD^+^ anabolism has been shown to ameliorate mitochondrial dysfunction in a mechanism dependent on SIRT1, a nuclear sirtuin.

While it is thought that NAD^+^ is predominantly an intracellular nucleotide, emerging evidence suggests that extracellular NAD^+^ crosses the plasma membrane and replenishes intracellular NAD^+^.^21^ Intracellular NAD^+^ concentrations have been shown to range between 10 and 1000 μM, and are much higher than the levels reported in the extracellular space.^22^ This is because (1) NAD^+^ is released from cells at low amounts; (2) NAD^+^ catabolism is rapid leading to biologically active products; and (3) NAD^+^ directly interacts with cell surface receptors such as connexion 43 channels and several subtypes of purinergic P2 receptors.^23^

Therefore, accurate monitoring of the plasma NAD^+^ metabolome is necessary and may provide valuable information regarding the effect of various lifestyle and dietary factors, pharmacological and nutraceutical supplementation of NAD^+^ and/or its metabolites. Monitoring the plasma NAD^+^ metabolome levels will also allow drug candidates to be screened for a new type of potentially adverse effect—the depletion of NAD^+^ and/or other desirable metabolites. Moreover, the ratio (e.g. the NAD:NADH ratio) of oxidized and reduced forms of pyridine dinucleotides provides important information regarding redox metabolism disorders or alterations to cellular bioenergetics and may become important biomarkers for the early detection of pathological states.

## Materials and Methods

### Participants

This human study was conducted in accordance with the ethical principles in the Declaration of Helsinki. This study was approved by the Institute of Regenerative and Cellular Medicine (IRCM) Institutional Review Board (IRB approval number IRCM-2016-128) according to the California Experimental Subject's Bill of Rights (California). Notice of authorization was granted on December 14, 2016 by the IRCM Institutional Review Board. The inclusion criteria were as follows: men and women between the ages of 20 and 87 with a body mass index (BMI) between 18 and 35 kg/m^2^ (±1 kg/m^2^). The patient characteristics by age cohort are shown in Table 1. Participants agreed to avoid taking vitamin B3 (NA, nicotinamide [NAM] or nicotinamide riboside [NR]) supplements or multivitamins for 14 days before this study and for the duration of the study period. Participants were considered “healthy” as determined by laboratory results, medical history, and physical examination.

**Table 1.** Patient Characteristics by Age Cohort

	20–40	41–60	60+
*N*	9	10	10
Age (SD)	22.78 (2.17)	52.80 (5.87)	76.90 (4.53)
BMI (SD)	20.38 (0.99)	22.80 (2.06)	22.25 (3.61)
Gender (M/F)	5/4	5/5	5/5

BMI, body mass index.

### Blood sample collection

Whole blood was collected in Vacutainer^®^ tubes containing 0.105 M buffered sodium citrate (BD Diagnostics, Franklin Lakes, NJ) as previously described.^24^ The tubes were gently inverted four times and centrifuged for 10 minutes at 2000 *g* using a refrigerated centrifuge. The collected plasma was transferred into a plastic Eppendorf tube and transferred to a −80°C freezer generally within 12 minutes after collection. Replicate aliquots were prepared and stored for each analysis to avoid the need for repeated freezing and thawing of the blood samples.

### Reagents, standards, and chromatography consumables

MS grade acetonitrile, AR grade formic acid, ammonium acetate (NH_4_OAc), ammonium hydroxide, and all metabolite standards were purchased from Sigma (Sydney, Australia). Isotopically enriched internal standards (IS), namely ^2^H_4_-NAM was purchased from Toronto Research Chemicals (Toronto, Canada). Three kiloDalton filters were purchased from Millipore (Melbourne, Australia). The amino phase (NH_2_) column was purchased from Phenomenex (Melbourne, Australia).

### Chromatographic separation of nucleotides and related metabolites and MS detection

Liquid chromatography coupled to tandem mass spectrometry (LC/MS/MS) was carried out using a Sciex QTRAP 5500 mass spectrometer (Sciex, Redwood City, CA) adapted from Bustamante et al.^25^ Briefly, 100 μL of human plasma was extracted in 400 μL of ice-cold methanol, centrifuged for 16.1 k*g* at 4°C for 10 minutes, and filtered through 3 kDa membrane cartridges. Sample extracts were dried under vacuum, reconstituted in 200 μL of 100 mM NH_4_OAc buffer and transferred into 200 μL glass vials and capped before LC/MS/MS analysis. Standards and samples (20 μL) were injected onto a Phenomenex NH_2_ column (150 mm × 2 mm × 3 μm) as previously described. A binary solvent gradient consisting of 5 mM NH_4_OAc pH 9.5 adjusted with ammonia (mobile phase A) and acetonitrile (mobile phase B) with a flow rate of 250 μL/min was used. Initial solvent composition at injection was 25% A, followed by a 2-minutes gradient to 45% A and a fast gradient ramp to 80% A (0.1 minutes) that was maintained for 5.9 minutes, A was increased again to 95% (2 minutes), held for 13 minutes, and then reverted to initial conditions (0.1 minutes) for equilibration, with a total run time of 30 minutes. The column flow was directed into the MS detector. Calibration curves of individual metabolites were constructed using the peak area ratios (peak area of the metabolite divided by peak area of the selected IS) of each calibrator versus its concentration. ^2^H_4_-NAM was used as the IS. The concentrations of the endogenous metabolites in the cell extracts were obtained from these calibration curves. Standard and sample chromatograms are shown as Supplementary Figures S1 and S2.

### Data analysis

All spectra were processed, and peak areas integrated using MultiQuant™ software (version 3.0, 2013; Sciex, Redwood City, MA). For groupwise comparisons, data are expressed as medians and IQR. Group variances were similar in all cases. A *p*-value <0.05 was considered significant. Analysis and plots were generated using RStudio with packages gplots, ggplots2, and corrplot. One-way analysis of variance (ANOVA) was used to compare means across age groups. Pearson's product-moment correlations were used to correlate levels of NAD^+^ metabolites with age. Correlation matrixes were ordered by hierarchical clustering to group together NAD^+^ metabolites with similar correlation patterns. Wilcoxon test was used to compare metabolite levels between sexes. Partial correlations were used to test the associations between BMI and metabolites correcting for age and sex. We corrected levels of statistical significance for multiple testing using the Benjamini-Hochberg false discovery method.

## Results

### Associations of NAD-related metabolites with age

**FIG. 1.**
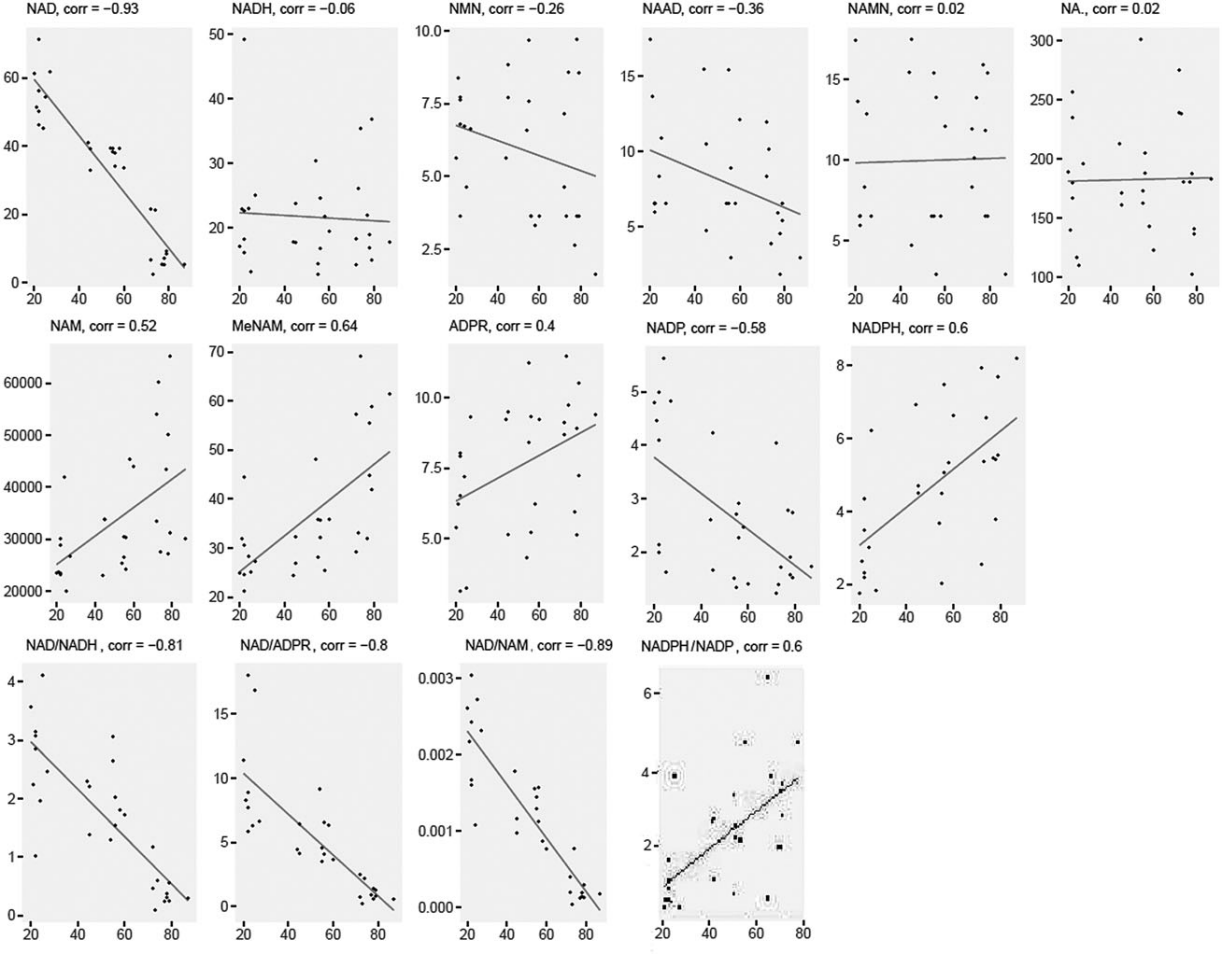
Scatterplots of NAD^+^ metabolites normalized abundance across age groups. NAD^+^, nicotinamide adenine dinucleotide. Concentrations are in nmol/L.

The NAD-related metabolites NAD^+^, NADP^+^ were significantly and negatively correlated with age from 20 to 87 years, while NAM, MeNAM, ADPR, and NADPH were significantly and positively correlated with age (all *r* > 0.30, Fig. 1). NAAD trended a negative association with age (*p* = 0.054), while NADH, nicotinamide mononucleotide (NMN), NAMN, and NA levels did not show significant changes with age (*p* > 0.05). Additionally, the ratios of NAD^+^:NADH, NAD^+^:ADPR, and NAD^+^:NAM were significantly decreased with age, while the NADPH:NADP^+^ ratio increased significantly with age (*p* < 0.05).

### Direct correlations of NAD-related metabolites with age

Direct correlation was applied to model changes to the NAD^+^ metabolome (Table 2) according to age. Most plasma NAD-related metabolite levels decreased with advanced age, with the exception of NMN, NAMN, and NA, which did not yield statistically significant changes (*p* > 0.05) while the levels of NAM, MeNAM, ADPR, and NAPDH increased with age (*p* < 0.05). NAD had the steepest age decrease, averaging 4% SD units decline per year, while MeNAM and NADPH had average increases of 2.8% and 2.7% SD units per year, respectively. The age of the donor explains to 86% of the variance in metabolite levels between individual samples.

**Table 2.** Ordinary Least Squares Regression Model of NAD^+^ Metabolite Abundance by Age

*Metabolite*	B Age^*^	r^2^	F	p	*SD*
NAD^+^	−0.040	0.86	167.2	4.4E-13	20.2
NAAD	−0.016	0.10	4.06	5.4E-02^a^	3.98
NAM	0.023	0.24	10.25	3.5E-03	11,829
MeNAM	0.028	0.39	18.88	1.7E-04	12.9
ADPR	0.017	0.13	5.22	3.0E-02	2.29
NADP^+^	−0.025	0.31	13.46	1.0E-03	1.33
NADPH	0.027	0.34	15.38	5.4E-04	1.97

^*^ metabolite abundances were standardized, so that B Age represents the slope in standard deviation (SD) units.

^a^ model trended towards significance *p* < 0.1.

ADPR, adenosine diphosphate ribose; MeNAM, N-methyl-nicotinamide; NAAD, nicotinic acid adenine dinucleotide; NAD+, nicotinamide adenine dinucleotide; NAM, nicotinamide.

### Mean differences by age group

**FIG. 2.**
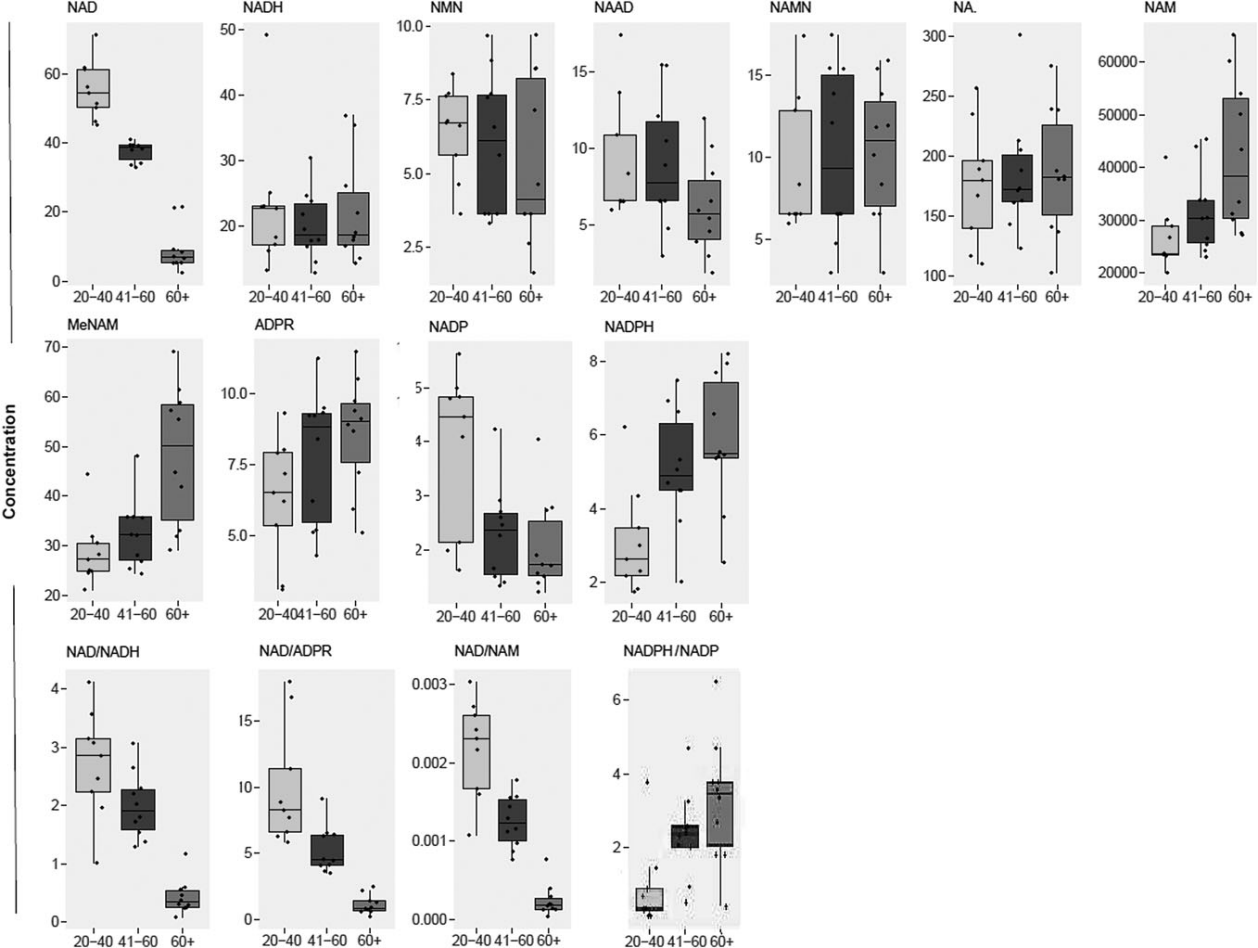
Boxplots of NAD^+^ metabolite abundances across age groups. Concentrations are in nmol/L.

Upon comparing abundances by age group (ANOVA), *post hoc* tests revealed that elderly subjects (60+ years) had significantly lower levels of NAD^+^, and NADP^+^ compared to young (20–40 years) subjects (Fig. 2). However, the levels of NAM, MeNAM, ADPR, and NADPH were significantly higher among elderly subjects compared with younger subjects. Some metabolites also appeared to show significant differences in plasma levels at middle age (41–60 years) compared with other age groups. During middle age, plasma NAD^+^ levels were significantly lower versus young subjects, and significantly higher compared with elderly subjects, while NADP^+^ and NADPH were significantly decreased and increased, respectively, compared to levels in young subjects. No significant differences were observed between age groups for NADH, NMN, NAMN, and NA (*p* > 0.05).

### Associations of NAD-related metabolites with gender

Using Wilcoxon test, NAAD was the only metabolite that trended toward significance (*p* < 0.10), with men having greater plasma levels than women (Supplementary Table S1).

### Associations of NAD-related metabolites with BMI

BMI did not correlate significantly with any metabolite after correcting for age and gender except for MeNAM (*r* = 0.597, *p* = 0.001) (Supplementary Table S2).

### Correlations of NAD^+^ metabolites to one another

**FIG. 3.**
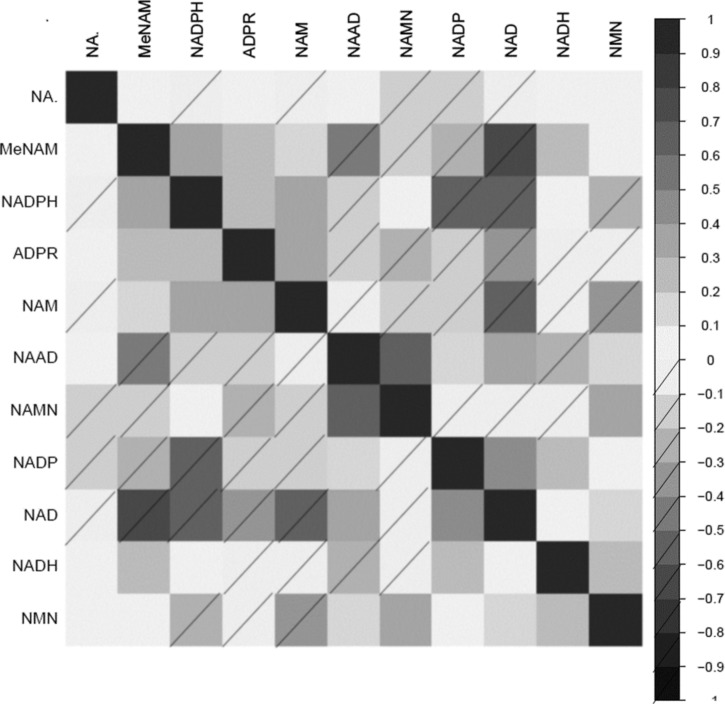
Correlation matrix of NAD^+^ metabolites with each other ordered by hierarchical clustering to group together the correlated NAD^+^ metabolite. Heatmap scale represents correlation strength, with *no dash* and *dash* for positive and negative correlations respectively.

Correlations of NAD-related metabolites to one another are shown in heatmap form (Fig. 3). ADPR was significantly and positively correlated with NAM, MeNAM, and NADPH. However, ADPR was negatively correlated with NAAD, NADP^+^, NAD^+^, NADH, and NMN. Most NAD-related precursors were positively correlated with NAD^+^, except for NAMN, which was not associated with NAD^+^, or NADH.

## Discussion

NAD^+^ was first discovered more than 110 years ago as a cofactor for fermentation processes. Afterward, NAD^+^ was later established as an important coenzyme involved in oxidation–reduction reactions necessary for oxidative phosphorylation and ATP production.^26^ More recently, numerous studies by our group and others have shown that NAD^+^ also interacts with a variety of specific proteins necessary to maintain health, including PARPs, NAD-dependent HDACs known as sirtuins, and NAD-glycohydrolases (CD38).^2–4,27^ These enzymes consume NAD^+^, and apart from metabolic processes such as glycolysis, the dissociation constants of these enzymes for NAD^+^ are within close range to physiological concentrations. We were the first to show that intracellular NAD^+^ levels decline with age in human skin tissue^20^ and catabolic organs in physiologically aged rats.^3^ Further, a recent study showed that the mRNA and protein expression of the connexin 43 hemichannel that mediates intracellular NAD^+^ influx in intact cells is reduced in retinal astrocytes with aging.^28^ This study supports and enhances this work showing that the levels of extracellular NAD^+^ metabolome are dysregulated in plasma collected from humans in multiple age groups using a modified LC-MS approach.

### The plasma NAD^+^/NADH ratio decreases with age

The balance between the amount of oxidized and reduced forms of NAD^+^ is known as the NAD^+^/NADH ratio, and represents a key measure of the cellular redox state, which reflects both total metabolic function, and the cellular health status.^29^ The NAD^+^/NADH ratio is regulated by several important enzymes including glyceraldehyde 3-phosphate dehydrogenase and pyruvate dehydrogenase.^30^ The physiological ratio of cytoplasmic NAD^+^ to NADH in mammalian tissue has been reported to be as high as 700, to favor oxidative phosphorylation and ATP production.^31^ However, we and others have shown that the NAD^+^/NADH ratio may vary between 0.01 and 5 in plasma.^32^

Our cross-sectional data show that plasma NAD^+^ levels are reduced among oldest subjects compared to middle aged subjects, which are in turn lower than young subjects. A lowered NAD^+^/NADH ratio has been shown to be associated with aging muscle and increased susceptibility to oxidative stress in catabolic organs.^33^ Although the exact mechanism of NAD^+^ decline remains unclear, it is likely that NAD^+^ levels are regulated by the rates of NAD^+^ synthesis, and NAD^+^ consumption.^34^ Our data suggest that it is primarily NAD^+^ that decreases with age to drive this ratio down, since NADH levels do not appear to be significantly changed across age groups. Additionally, there may be other causes of NAD^+^ decline, including a metabolic shift that does not favor higher NAD^+^/NADH ratio.^33^ Therefore, age-related metabolic impairments may not be entirely due to the accumulation of oxidative damage to macromolecules. Rather, these abnormalities may be due to a reduction in the levels of key pyridine nucleotides, and NAD^+^ in particular, which research shows can be reversed.

### The plasma NADPH/NADP^+^ ratio increases with age

It has been previously reported that the NADPH/NADP^+^ ratio is ∼1.05 in whole cells isolated from *Saccharomyces cerevisiae*.^35^ NADPH is produced by several reactions including glucose-6-P dehydrogenase (G6PDH) and 6-phosphogluconate dehydrogenase (6PGDH) of the Pentose Phosphate Pathway (PPP).^35^ While the reactions involved in the production of NADPH have a high thermodynamic drive, reactions that consume NADPH are active close to equilibrium.^36^ Elevations in the NADPH/NADP^+^ ratio are necessary for increased production of numerous molecules, including fatty acids, amino acids, and some products of the mevalonic acid pathway.^35^ In mammalian cells, fatty acids are essential for energy production. The primary reaction that facilitates the production of fatty acids involves the reduction of acetyl-CoA and CO2 to malonyl-CoA in the cytoplasm, using NADPH as the essential cofactor. Increased NADPH/NADP^+^ represents a key regulator of fatty acid synthesis,^37^ which in turn can impair amino acid production. For instance, the levels of the amino acids, l-arginine, and l-lysine, have been correlated to the availability of NADPH.^38^ There are also several other reactions that are dependent on high levels of NADPH. For example, reduction reactions involving toxic aldehyde intermediates (which accumulate with age) are regulated by the levels of NADH and NADPH.^39^ In these processes, the aldehyde is reduced to alcohol using NADH and NADPH.

On the contrary to previous studies, our data suggest that while it is likely that oxidative stress increases with age, the NADPH/NADP^+^ paradoxically increases with age. In aerobic organisms, the synthesis of NADH and NADPH is dependent on processes that convert NAD^+^ into NADP^+^. The synthesis of NADP^+^ from NAD^+^ is catalyzed by NADkinases (NADK), and the transformation of NADP^+^ to NAD^+^ is regulated by NADPases. By altering the levels of NAD^+^ and NADP^+^, these enzymes can influence the concentrations of NADPH and NADH. It has been previously hypothesized that reduced levels of NAD^+^ may impair NADH production as a compensatory mechanism to lower cellular oxidative stress levels, since the generation of volatile free radicals as a consequence of oxidative phosphorylation is ameliorated.^40^ Therefore, the activity of NADK and NADPase may represent important effectors of the cellular redox state. It is likely that upregulation of NADK and the downregulation of NADPase during the aging process may enhance the production of NADPH and decrease the formation of NADH to lower oxidative stress. Further work is needed to confirm whether the levels of NAD^+^ and the NADPH/NADP^+^ ratio are regulated by age-related changes to the activity and/or expression of NADK and NADPase following accumulation of oxidative stress.

### Is NAD^+^ decline due to increased NAD^+^ turnover or reduced NAD^+^ synthesis?

It remains unclear why the catabolism and synthesis of NAD^+^ do not stay in balance as organisms age, but instead the synthesis appears to become outpaced by consumption.

Our data show that the plasma levels of the NAD^+^ precursor NAAD was reduced with advanced aging. Increased overexpression of major pathways in the NAD^+^ salvage pathway have been shown to increase NAD^+^ levels and extend lifespan in small organisms and murine models.^41^ Additional studies using NAD^+^ precursors such as NR or NMN to increase NAD^+^ levels and improve mitochondrial function suggests that the NAD^+^ salvage pathway may be impaired up to or including the reaction mediated by nicotinamide phosphoribosyltransferase (NAMPT), which generates NMN as a product.^42,43^ Overexpression of NAMPT has been shown to restore mitochondrial function, suggesting that NAMPT is the rate-limiting enzyme for optimal NAD^+^ production from NAM.^44^ Further studies are needed to determine whether other upstream NAD^+^ precursors, such as NAM, NA, and NMN are more efficient to increase NAD^+^ levels to a similar extent as NR.

Other possibilities include increased NAD^+^ catabolism via increased hydrolysis or NAD^+^ polymerization by the DNA nick sensor, PARP. Sirtuins can also break down NAD^+^ in deacetylation reactions. CD38 are NAD-dependent glycohydrolases that can also break down NAD^+^ and have been associated with immune responses and transient intracellular calcium influx. Consistent with our current findings, we have previously shown that DNA damage correlated positively with increased PARP activity and NAD^+^ decline in catabolic organs from physiologically aged rats and human pelvic skin.^3,4,20^ Our current data show that ADPR, which is a product of PARP activity, is increased in human plasma after middle age. Another study previously showed that PARP activity increased in rat glial and neuronal cell cultures isolated from aged rats compared with young rats,^45^ and PARP activity was increased in mammalian leukocytes, and cells isolated from human centenarians. Increased levels of ADPR have also been previously reported in the temporal and frontal cortex in the brains of Alzheimer's patients compared with controls.^46^ While low to moderate PARP activity may play a protective role,^47^ increased PARP activity may deplete cellular NAD^+^ concentrations. Increased PARP activity can lead to reduced SIRT1 activity, which is required for TFAM expression and mitochondrial oxidative phosphorylation.

CD38 also appears to be a major consumer of NAD^+^ during the aging process. Given that 100 molecules of NAD^+^ must be hydrolyzed to generate one molecular of cADPR, it is highly likely that CD38 is a major regulator of intracellular NAD^+^ levels.^48^ Accordingly, we previously found a fivefold increase in NAD^+^ levels in CD38 knockout neuronal cells compared with controls.^4^ Therefore, CD38 may not only represent an inefficient secondary messenger enzyme, but also as an NADase that primarily regulates intracellular levels of NAD^+^ and its physiological processes. Inhibition of CD38 using the natural phytochemical inhibitor, apigenin increased NAD^+^ levels and protected against degeneration in mice exposed to a high-fat diet.^49^ Interestingly, CD38 has been shown to degrade NAD^+^ and its precursor NMN *in vivo*.^50^ One study showed that the plasma levels of NAD^+^ remained stable when CD38 knockout mice were administered intraperitoneal injections of NAD^+^, NMN, or NR after 3 hours, long after they began to fall in the wild-type animals.^51^ This suggests that the efficacy of NAD^+^ precursors may be enhanced by combination therapy with CD38 inhibitors such as apigenin.

### Accumulation of MeNAM—a double-edged sword

In mammalian cells, NAM is methylated by the enzyme nicotinamide N-methyltransferase (NNMT) to form MeNAM, which is further metabolized to N-methyl-2-pyridone-5-carboxamide (2PY).^52^ Increased levels of NAM have been shown to stimulate NAD^+^ synthesis by entering the polyamine flux.^53^ One study showed that knockdown to NNMT protected against high fat diet-induced obesity.^54^ Another study further showed that undernourished children excreted higher amounts of MeNAM and 2-PY than in normal children.^55^ This may represent a metabolic adaptation to enhance NNMT activity to lower NAM levels and promote energy expenditure. Similarly, children with better growth excreted lower amounts of MeNAM and 2-PY.^55^ Therefore, it has been suggested that better growers could adapt better than poor growers to a nutritionally limited environment by lowering energy expenditure in favor of growth.

Our data also show that the level of MeNAM was correlated with BMI (Supplementary Table S2), and MeNAM has been strongly associated with obesity and diabetes.^56^ Therefore, a higher level of MeNAM is likely to predispose individuals to a greater risk of obesity or insulin resistance in adulthood. Increased NNMT activity and elevations in MeNAM and 2-PY levels have also been reported in type-2 diabetes.^57^ Increased NNMT activity in tumor cells has been shown to impair polyamine flux.^58^ Therefore, increased NNMT activity and upregulated levels of MeNAM can alter individual methylation capacity with epigenetic consequences.

Recently, it has been shown that metabolites of NAM may serve as uremic toxins at high amounts.^59^ These findings originated from findings of interventional trials using NAM to manage end-stage renal disease.^60^ In the context of uremia, increased plasma levels of MeNAM, as we have reported, may inhibit NAD-dependent processes. Our current findings can therefore provide additional mechanisms to explain poor outcomes in the elderly, and the potential effects of impaired NAD^+^ anabolism on the epigenome due to potentially reduced methylation capacity in the aged individuals.

### Limitations

While our study provides a good overview of the changes to the extracellular plasma NAD^+^ metabolome with age, NAAD was the only metabolite that shows a greater significance in men compared with women. In a recent study comparing three NAD^+^ precursor vitamins provided in bolus at equivalent oral doses, NAAD was reported to correlate well with increased NAD^+^ synthesis.^19,56^ However, it remains unclear whether the rate of NAD^+^ synthesis is greater in men than women in this study, since no significant difference in other metabolites including NAD^+^ was observed between men and women (Supplementary Table S1). There are also several limitations: (1) we focused on subjects aged between 20 and 85 years, which prevents direct comparisons with children and prepubescent teens; (2) a largely Caucasian population in the United States of America was used; as the NAD^+^ metabolome is regulated by several dietary and lifestyle factors, the association of our findings should be interpreted with caution in non-Caucasian subjects^61^; (3) our sample size of 30 subjects, split by sex, is relatively small for detailed correlations, even among gender. Ideally, a larger number of subjects in the order of several hundred subjects are favorable to increase significance; and (4) the present study uses a cross-sectional design. To gain a greater understanding of intra-individual variation in the NAD^+^ metabolome with age, additional longitudinal studies should be performed.

## Conclusion

To our knowledge, this is the first study to show that NAD^+^ levels steeply declines with age in human plasma. On the contrary to previous reports, this study provides strong evidence for the availability of the extracellular NAD^+^ and its related metabolites in plasma. This is important since NAD^+^ is vital to numerous cellular processes including oxidative phosphorylation and energy production, immune function (through CD38 activity), DNA repair (through PARP activity), nuclear signaling and protein function (through sirtuin activity), and telomere maintenance (through Tankyrase1 activity). Critical depletion of NAD^+^ results in cell death through reduced ATP production and activation of apoptosis.^62^ Reliable measurements of plasma NAD^+^ metabolites should greatly aid in the effective upregulation of intracellular NAD^+^ using NAD^+^ and related precursors for the purpose of maintaining critical cellular processes and thereby ameliorating some age-related cellular degeneration and resulting pathologies.

## Supplementary Material

Supplemental data

Supplemental data

Supplemental data

Supplemental data
