# Supplementary material for: The Plasma NAD+ Metabolome Is Dysregulated in “Normal” Aging
Source: Rejuvenation Res. 2019 Apr 23;22(2):121–30. doi: 10.1089/rej.2018.2077 (PMC6482912; doi:10.1089/rej.2018.2077)
Supplement: Supplemental data [file Supp_Fig1.pdf]

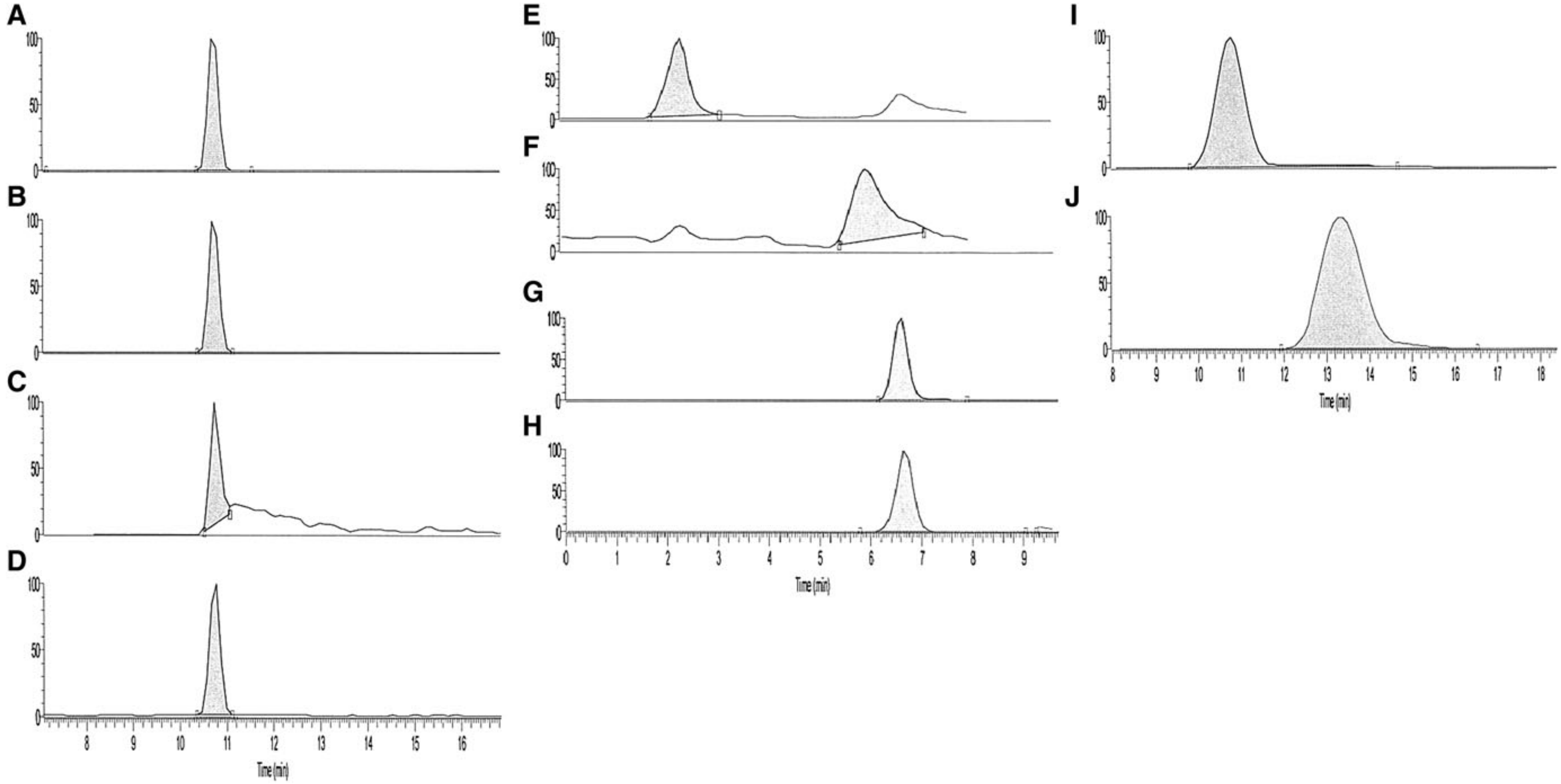

SUPPLEMENTARY FIG. S1. Chromatograms of metabolites of standards sample. **(A)** NAD+; **(B)** NADH; **(C)** ADPR; **(D)** NAMN; **(E)** NAM; **(F)** NA; **(G)** NMN; **(H)** NAAD; **(I)** NADP+; **(J)** NADPH.
