# Supplementary material for: The Plasma NAD+ Metabolome Is Dysregulated in “Normal” Aging
Source: Rejuvenation Res. 2019 Apr 23;22(2):121–30. doi: 10.1089/rej.2018.2077 (PMC6482912; doi:10.1089/rej.2018.2077)
Supplement: Supplemental data [file Supp_Fig2.pdf]

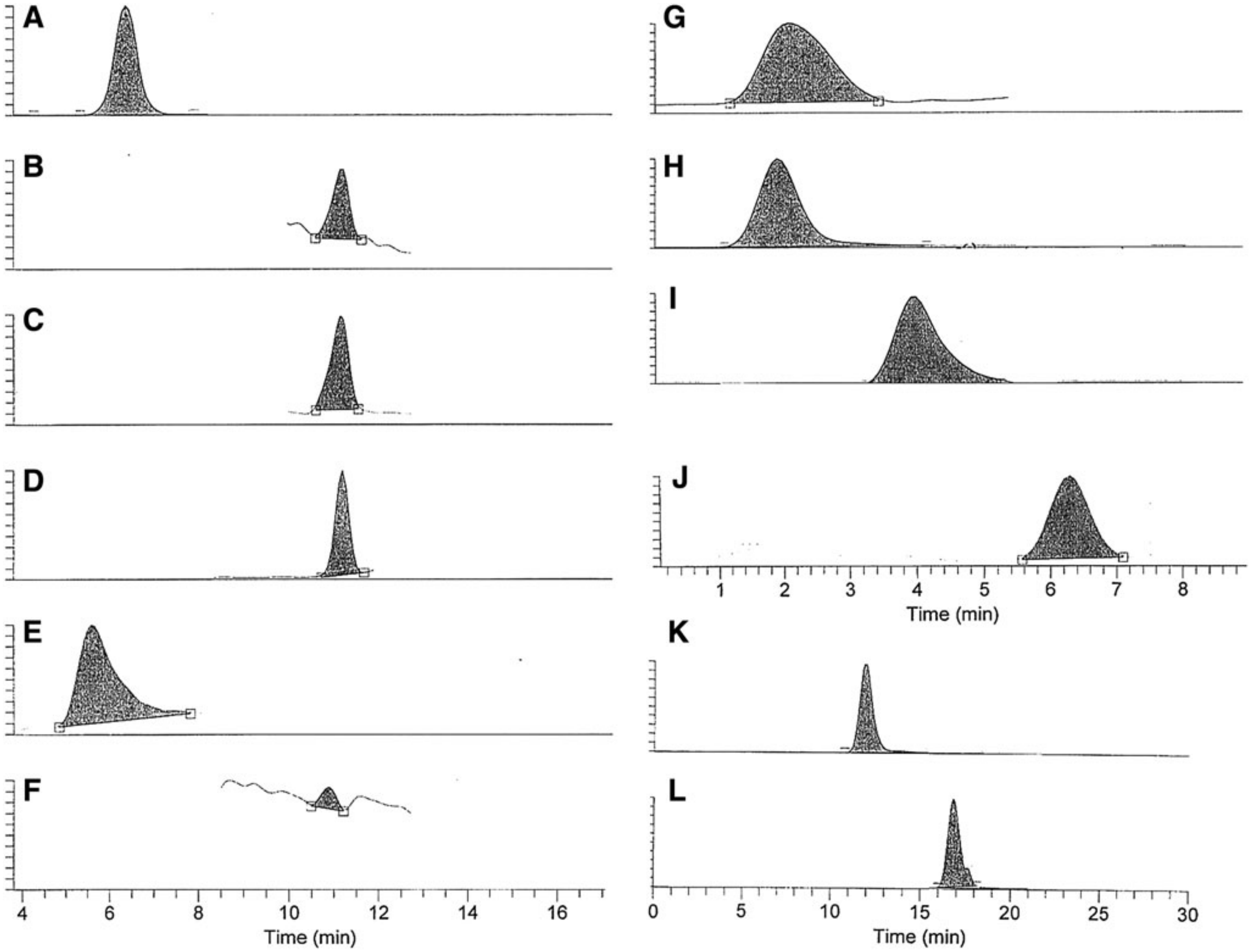

SUPPLEMENTARY FIG. S2. Chromatograms of metabolites in sample. **(A)** NAD+; **(B)** NAAD; **(C)** NADH; **(D)** ADPR; **(E)** NA; **(F)** NAMN; **(G)** NAM; **(H)** d4-NAM; **(I)** MeNAM; **(J)** NMN; **(K)** NADP+; **(L)** NADPH.
