# Supplementary material for: The Plasma NAD+ Metabolome Is Dysregulated in “Normal” Aging
Source: Rejuvenation Res. 2019 Apr 23;22(2):121–30. doi: 10.1089/rej.2018.2077 (PMC6482912; doi:10.1089/rej.2018.2077)
Supplement: Supplemental data [file Supp_Table1.pdf]

Supplementary Table S1. Associations of NAD-Related Metabolites with Gender

	NAD+	*NADH*	*NMN*	*NAAD*	*NAMN*	*NA*	*NAM*	*MeNAM*	*ADPR*	*NADP*	*NADPH*	*NAD+:NADH*	*NAD+:ADPR*	*NAD+:NAM*
Mann–Whitney *U*	87.000	82.000	100.000	61.000	79.500	91.000	89.000	100.000	95.000	88.000	89.000	94.000	88.000	88.000
Wicoxon W	192.000	187.000	205.000	152.000	184.500	196.000	209.000	220.000	215.000	208.000	209.000	199.000	193.000	193.000
Z	–0.786	–1.004	–0.220	–1.702	–1.130	–0.611	–0.698	–0.218	–0.436	–0.742	–0.698	–0.480	–0.742	–0.742
Asymp. sig. (two-tailed)	0.432	0.315	0.826	0.089	0.258	0.541	0.485	0.827	0.663	0.458	0.485	0.631	0.458	0.458
Exact sig. [two* (one-tailed sig.)]	0.451[b]	0.331[a]	0.847[a]	0.098[a]	0.270[a]	0.561[a]	0.505[a]	0.847[a]	0.683[a]	0.477[a]	0.505[a]	0.652[a]	0.477[a]	0.477[a]

[a]Not corrected for ties.

ADPR, adenosine diphosphate ribose; MeNAM, N-methyl-nicotinamide; NA, nicotinic acid; NAAD, nicotinic acid adenine dinucleotide; NAD+, nicotinamide adenine dinucleotide; NAM, nicotinamide; NAMN, nicotinic acid mononucleotide; NMN, nicotinamide mononucleotide.
