# Supplementary material for: The Plasma NAD+ Metabolome Is Dysregulated in “Normal” Aging
Source: Rejuvenation Res. 2019 Apr 23;22(2):121–30. doi: 10.1089/rej.2018.2077 (PMC6482912; doi:10.1089/rej.2018.2077)
Supplement: Supplemental data [file Supp_Table2.pdf]

Supplementary Table S2. Associations of NAD-related Metabolites with BMI

Control variables (age and gender)	*Statistical analysis*	*BMI*
BMI	Correlation	1.000
	Significance (two-tailed)	0.000
	df	24
NAD+	Correlation	0.071
	Significance (two-tailed)	0.732
	df	24
NADH	Correlation	0.162
	Significance (two-tailed)	0.429
	df	24
NMN	Correlation	0.023
	Significance (two-tailed)	0.910
	df	24
NAAD	Correlation	0.005
	Significance (two-tailed)	0.980
	df	24
NAMN	Correlation	0.216
	Significance (two-tailed)	0.290
	df	24
NA	Correlation	–0.086
	Significance (two-tailed)	0.676
	df	24
NAM	Correlation	0.125
	Significance (two-tailed)	0.543
	df	24
MeNAM	Correlation	0.597
	Significance (two-tailed)	0.001
	df	24
ADPR	Correlation	–0.204
	Significance (two-tailed)	0.318
	df	24
NADP+	Correlation	–0.216
	Significance (two-tailed)	0.289
	df	24
NADPH	Correlation	–0.149
	Significance (two-tailed)	0.467
	df	24
NAD+:NADH	Correlation	0.102
	Significance (two-tailed)	0.621
	df	24
NAD+:ADPR	Correlation	0.052
	Significance (two-tailed)	0.799
	df	24
NAD+:NAM	Correlation	–0.043
	Significance (two-tailed)	0.834
	df	24

BMI, body mass index.
